# Botanical, Pharmacological, Phytochemical, and Toxicological Aspects of the Antidiabetic Plant *Bidens pilosa* L.

**DOI:** 10.1155/2014/698617

**Published:** 2014-01-29

**Authors:** Wen-Chin Yang

**Affiliations:** ^1^Agricultural Biotechnology Research Center, Academia Sinica, No. 128, Academia Sinica Road, Section 2, Nankang, Taipei 115, Taiwan; ^2^Institute of Pharmacology, Yang-Ming University, Taipei 112, Taiwan; ^3^Department of Life Sciences, National Chung-Hsing University, Taichung 402, Taiwan; ^4^Institute of Zoology, National Taiwan University, Taipei 106, Taiwan; ^5^Department of Aquaculture, National Taiwan Ocean University, Keelung 202, Taiwan

## Abstract

*Bidens pilosa* L. is an easy-to-grow, widespread, and palatable perennial on earth. Hence, it has traditionally been used as foods and medicines without noticeable adverse effects. Despite significant advancement in chemical and biological studies of *B. pilosa* over the past few years, comprehensive and critical reviews on its anti-diabetic properties are missing. The present review is to summarize up-to-date information on the pharmacology, phytochemistry, and toxicology of *B. pilosa*, in regard to type 1 diabetes and type 2 diabetes from the literature. In addition to botanical studies and records of the traditional use of *B. pilosa* in diabetes, scientific studies investigating antidiabetic action of this species and its active phytochemicals are presented and discussed. The structure and biosynthesis of *B. pilosa* and its polyynes in relation to their anti-diabetic action and mechanism are emphasized. Although some progress has been made, rigorous efforts are further required to unlock the molecular basis and structure-activity relationship of the polyynes isolated from *B. pilosa* before their clinical applications. The present review provides preliminary information and gives guidance for further anti-diabetic research and development of this plant.

## 1. Introduction

Diabetes was coined by a Greek physician, Aretaeus of Cappadocia (30-90 AD), about 2 millennia ago [1]. He first described this devastating disease with symptoms, such as constant thirst (polydipsia), excessive urination (polyuria), and weight loss, which still hold true nowadays [1]. The International Diabetes Federation (IDF) estimated that diabetes afflicted 285 million people, 6.4% of the world population, who were afflicted with diabetes in 2010 and will afflict 439 million people, 7.7% of the world population by 2030 [2]. Over 90% of diabetic patients are diagnosed with type 2 diabetes (T2D) [3, 4] and the rest are diagnosed with type 1 diabetes and others.

Diabetes is a chronic metabolic disease with fatal complications such as cardiovascular diseases, retinopathy, renopathy, and foot ulcers. The cost of health care associated with diabetes continues to grow and is a huge economic burden for afflicted patients and countries. In the states, approximately 17.5 million adults were reported to be receiving treatment for diabetes, where the estimated cost of diabetes was 174 billion dollars in 2007 [5].

Despite much progress made on basic and clinical research into diabetes, this disease has not been cured since antiquity. Main reasons for this mishap are unmet efficacy and significant side effect of the drugs. So far, 1200 plants have been claimed to be remedies for diabetes [6, 7] and one-third of them have been scientifically evaluated for T2D treatment [8]. Among them, *B. pilosa* is commonly used as food and medicine for humans and animals [9, 10]. It is an easy-to-grow herb that is globally distributed. The folkloric use of *B. pilosa* to treat diabetes has been recorded in America, Africa, Asia, and Oceania [11]. Accumulating data have shown the potential of this plant and active compounds to treat diabetes. The present review focuses on recent studies on the botany, anti-diabetic action and mechanism, phytochemistry, and toxicology of *B. pilosa*. The information provided here highlights the possible usefulness of *B. pilosa* and its isolated compounds and offers insights into possible future research directions.

## 2. Botanical Properties

**Figure 1:**
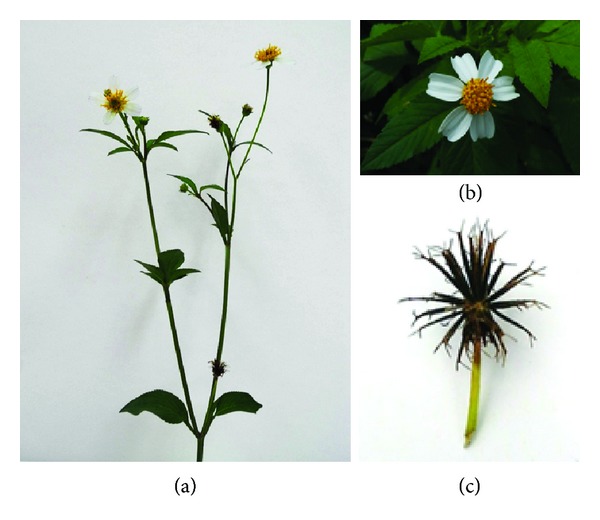
Image of *B. pilosa *(a) and its flowers (b) and seeds (c).

**Figure 2:**
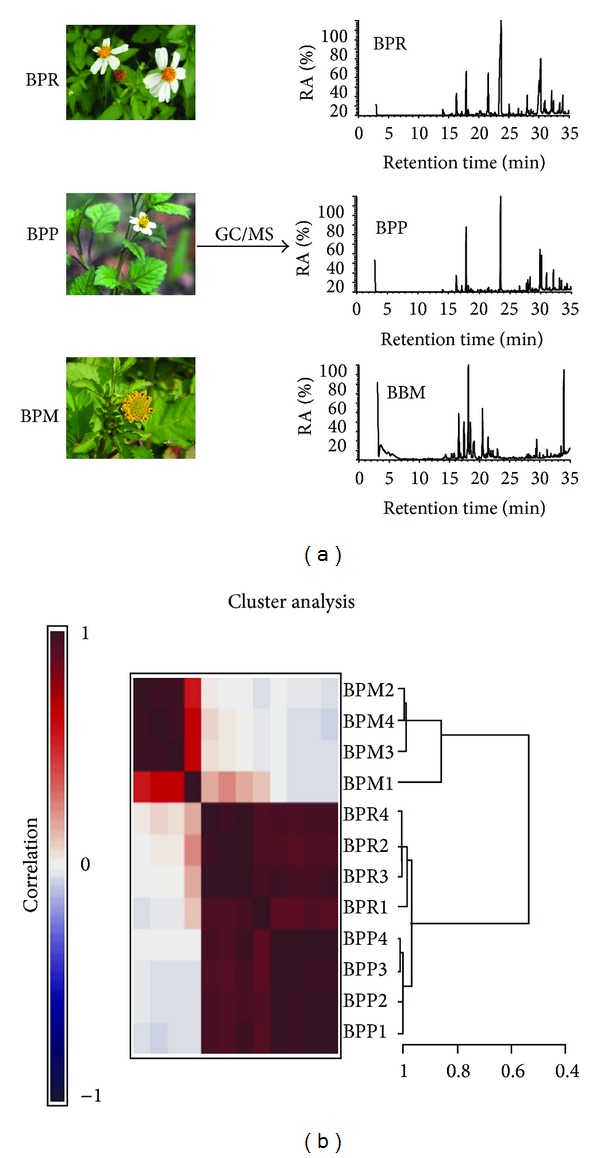
Chemotaxonomical comparison of three *B. pilosa *variants (BPR, *B. pilosa *var. *radiata*; BPM, *B. pilosa *var. *Minor,* and BPP, *B. pilosa *var. *pilosa*). Gas chromatography/mass spectrometry (GC/MS) and cluster analysis to assist in determining the taxonomy of 4 samples of the three *Bidens* variants.

*B. pilosa *is believed to have originated in South America and subsequently spread everywhere on earth [13]. *Bidens *species and their varieties bear vernacular names based on their sticky seeds or prosperous growth [5]. *B. pilosa* is taxonomically assigned to the *Bidens *genus, up to 240 species, as shown in Table 1 [9, 10]. Different varieties are frequently found in *B. pilosa*. *B. pilosa* is an erect, perennial herb widely distributed from temperate and tropical regions. It has serrate, lobed, or dissected form of green opposite leaves, white or yellow flowers, and long narrow ribbed black seeds (Figure 1) [14]. Apart from morphological traits, chemotaxonomical (Figure 2) and molecular characterization (Table 2) is sometimes helpful in the identification of *B. pilosa *strains [15].

Despite its preference for full sun and semidry soil,* B. pilosa *can grow in arid and barren lands at different altitudes. Food and Agricultural Organization actively promoted the culture of *B. pilosa* in Africa in 1970s due to its fast-growing advantage [16]. *B. pilosa* can be propagated via seeds. After soaking, *B. pilosa* seeds can germinate in 3 to 4 days [17]. Minimal agricultural techniques are required for *B. pilosa* cultivation. *B. pilosa *is recognized as one of the top worst weeds worldwide because of its aggressive invasion [18].

Apart from its use as food ingredient, *B. pilosa *is used as herbal medicines for diabetes and 40 other diseases [5]. All parts of *B. pilosa* plant, the whole plant, the aerial parts (leaves, flowers, seeds, and stems), and/or the roots, fresh or dried, are used as ingredients in folk medicines. Dry powder, decoction, maceration, and tincture are usual formulations for its internal as well as external use [19]. *B. pilosa* can be used alone or together with other medicinal herbs.

## 3. Antidiabetic Properties

*B. pilosa* has a variety of pharmacological actions. As far as its anti-diabetic activity is concerned, *B. pilosa *and its anti-diabetic polyynes have been reported to effectively prevent and treat type 1 diabetes and type 2 diabetes, which are etiologically distinct [15, 20–24]. In this section, we will focus our review on the pharmacological action and mechanism of *B. pilosa* extract and its active phytochemicals against both types of diabetes.

### 3.1. Action and Mechanism of *B. pilosa* for Type 1 Diabetes

T1D is caused by the autoimmune destruction of pancreatic *β* cells, leading to insulin deficiency, hyperglycemia, and complications. Monotherapy (immune intervention and *β*-cell replacement/(re)generation) and their combination therapy are common approaches to treat T1D. Despite considerable advances made on these approaches, there has no cure for T1D. Helper T (Th) cell differentiation regulates T1D development. Further, Th1 cell differentiation promotes T1D, whereas Th2 cell differentiation alleviates T1D [25].

**Figure 3:**
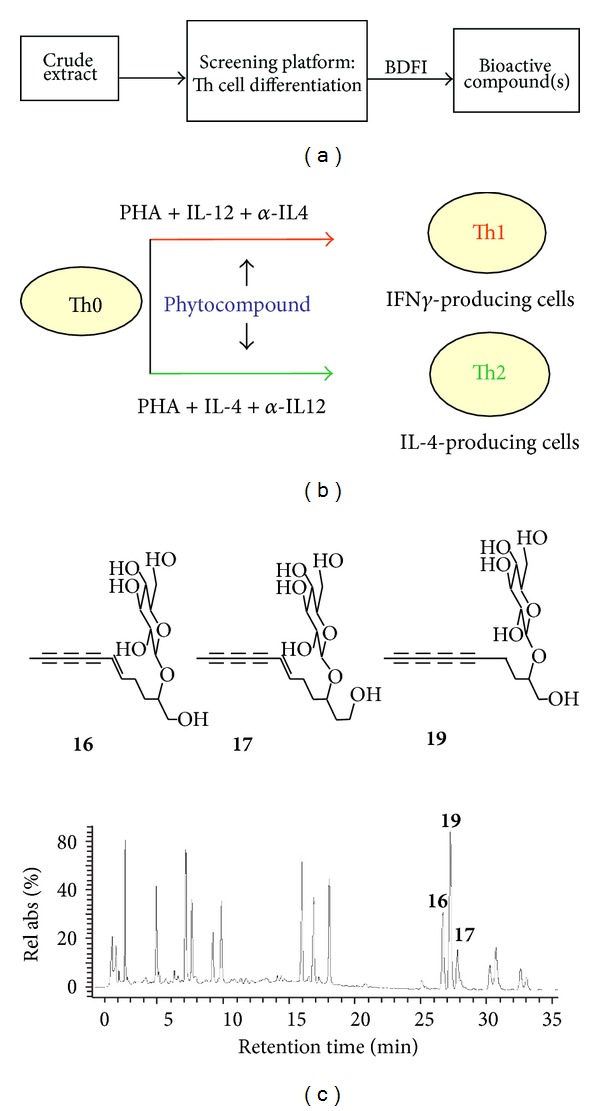
Bioactivity-directed fractionation and isolation approach to identify three active polyynes that regulate Th cell differentiation. A flowchart of the bioactivity-directed fractionation and isolation (BDFI) strategy describes the use of the screening platform and Th cell differentiation assays to determine bioactive compounds from the crude extract and fractions of *B. pilosa* (a). Bioassays are composed of human naïve helper T cells (Th0) which can differentiate into type 1 helper T (Th1) cells and type 2 helper T (Th2) cells in the presence of PHA plus IL-12 and anti-IL-4 antibody and PHA with IL-4 and anti-IL-12 antibody, respectively. The crude extract, fractions, and compounds of *B. pilosa* are added to differentiating cells to test the Th cell differentiation (b). Compounds **16**, **17**, and **19** are active compounds that promote Th2 cell differentiation but inhibit Th1 cell differentiation.

To test the immunomodulatory effect of *B. pilosa*, one study showed that *B. pilosa* extract and its butanol fraction could decrease Th1 cells and cytokines and increase Th2 cells and cytokines [23]. This study indicated that IC_50_ value of the butanol fraction was 200 *μ*g/mL. This inhibition was reported to be partially attributed to cytotoxicity, because the butanol fraction at 180 *μ*g/mL could cause 50% death of Th1 cells. Using the bioactivity-directed isolation and identification approach (Figure 3), 3 active polyynes, 3-*β*-D-glucopyranosyl-1-hydroxy-6(*E*)-tetradecene-8,10,12-triyne (**17**), 2-*β*-D-glucopyranosyloxy-1-hydroxy-5(*E*)-tridecene-7,9,11-triyne (**16**), and 2-*β*-D-glucopyranosyloxy-1-hydroxytrideca-5,7,9,11-tetrayne (cytopiloyne,** 19**), as well as 2 index compounds, 4,5-Di-*O*-caffeoylquinic acid, 3,5-Di-*O*-caffeoylquinic acid, and 3,4-Di-*O*-caffeoylquinic acid, were isolated from the butanol extract using high pressure liquid chromatography (HPLC) and, in turns, were structurally identified by nuclear magnetic resonance (NMR) [23, 24]. Only the first three active compounds showed similar effects on Th cell differentiation as the *B. pilosa *butanol fraction. Moreover, compound **19** showed greater activity than compounds **17 **and **16** in terms of enhancement (by 277% compared to 34% and 8%) of differentiation of Th0 to Th2 at 10 *μ*g/mL and inhibition (by 60% compared to 17% and 9%) of differentiation to Th1 at the same concentration (Table 3) [23, 24].

Accordingly, the butanol fraction of *B. pilosa* effectively prevented T1D in nonobese diabetic (NOD) mice [23]. Consistently, this prevention involved downregulation of Th1 cells or upregulation of Th2 cells. This was proven by intraperitoneal injection of the butanol fraction at a dose of 3 mg/kg body weight (BW), 3 times a week, to NOD mice from 4 to 27 weeks [23]. This dosage resulted in lower incidence of diabetes (33%). At a dose of 10 mg/kg, the butanol fraction of *B. pilosa* totally stopped (0%) the initiation of the disease [23]. Th1 cytokine IFN*γ* and Th2 cytokine IL-4 favor the production of IgG2a and IgE, respectively. To further confirm whether this butanol *in vivo* regulated Th cell differentiation and Th cytokine profiling, IgG2a and IgE production was measured in the serum of NOD mice. As expected, high levels of IgE and some decline in the levels of IgG2a were observed in the serum [23].

Since cytopiloyne (**19**) had the most potent effect on Th cell differentiation among the aforesaid polyynes [20], another study used cytopiloyne to explore the action and molecular mechanism of cytopiloyne on T1D in NOD mice [20]. NOD mice received intraperitoneal or intramuscular injection of cytopiloyne at 25 *μ*g/kg BW, 3 times per week. Twelve-week-old NOD mice started to develop T1D, and 70% of NOD mice aged 23 weeks and over developed T1D. Remarkably, 12- to 30-week-old NOD mice treated with cytopiloyne showed normal levels of blood glucose (<200 mg/dL) and insulin (1-2 ng/mL). Consistent with T1D incidence, cytopiloyne delayed and reduced the invasion of CD4^+^ T cells into the pancreatic islets [20]. Albeit less effective than cytopiloyne (**19**), 3-*β*-D-glucopyranosyl-1-hydroxy-6(*E*)-tetradecene-8,10,12-triyne (**17**), and 2-*β*-D-glucopyranosyloxy-1-hydroxy-5(*E*)-tridecene-7,9,11-triyne (**16**) also decreased T1D development in NOD mice.

**Figure 4:**
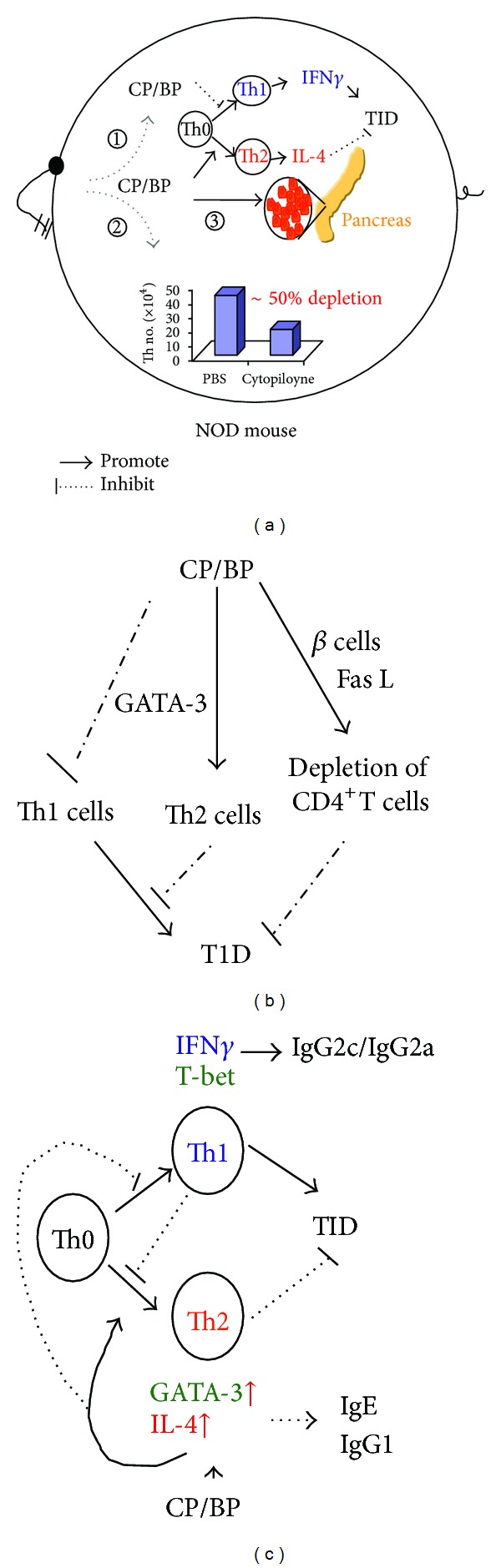
The underlying mechanism of the crude extract of *B. pilosa* (BP) and its active compound, compound **19** (CP), in T1D. BP and/or CP can suppress T1D development via regulation of T cells (*①* and *②*) and *β* cells (*③*) in NOD mice (a). Their regulation of T cells involves Th cell activation and differentiation (*①*) and partial depletion of Th0 cells (*②*) as depicted (b). CP and/or BP augment the expression of GATA-3 gene and, in turn, promote the expression of IL-4 and Th2 cell differentiation. In contrast, CP and/or BP do not affect the expression of T-bet (c).

The underlying mechanism by which cytopiloyne and its derivatives inhibited T1D covered inactivation of T cells, polarization of Th cell differentiation, and Th cell depletion, leading to islet protection [20] and is illustrated in Figure 4. First, [^3^H] thymidine incorporation assay showed that cytopiloyne inhibited ConA/IL-2- and CD3 antibody-mediated T cell proliferation, implying that cytopiloyne could inhibit T cell activation. Second, *in vitro* study showed that cytopiloyne (**19**) inhibited the differentiation of naïve Th (Th0) cells (i.e., CD4^+^ T cells) into Th1 cells and promoted differentiation of Th0 cells into Th2 cells [24]. The *in vitro* data are consistent with the *in vivo* results, indicating that cytopiloyne reduced Th1 differentiation and increased Th2 differentiation as shown by intracellular cytokine staining and FACS analysis [20]. Cytopiloyne also enhanced the expression of GATA-3, a master gene for Th2 cell differentiation, but not the expression of T-bet, a master gene for Th1 cell differentiation, further supporting its role in skewing Th differentiation [20]. In line with the skewing of Th differentiation, the level of serum IFN-*γ* and IgG2c decreased, while that of serum IL-4 and serum IgE increased compared to the negative controls (PBS-treated mice). Third, cytopiloyne partially depleted CD4^+^ rather than CD8^+^ T cells in NOD mice [20]. Coculture assays showed that the depletion of CD4^+^ T cells was mediated through the induction of Fas ligand expression on pancreatic islet cells by cytopiloyne, leading to apoptosis of infiltrating CD4^+^ T cells in the pancreas via the Fas and Fas ligand pathway. However, cytopiloyne did not induce the expression of TNF-*α* in pancreatic islet cells and, thus, had no effect on CD8^+^ T cells [20].

Due to the antidiabetic mechanisms of action, it was hypothesized that cytopiloyne protects NOD mice from diabetes by a generalized suppression of adaptive immunity. To evaluate this hypothesis, ovalbumin (Ova) was used as a T-cell dependent antigen to prime NOD mice, which had already received cytopiloyne or PBS vehicle. Ova priming enhanced similar anti-Ova titers in cytopiloyne-treated mice and PBS-treated mice, but a different profile of immunoglobulin isotype was observed in the two groups. Therefore, it was concluded that cytopiloyne is an immunomodulatory compound rather than an immunosuppressive compound [20, 24].

Collectively speaking, the mechanism of action of cytopiloyne and, probably, its polyyne derivatives in T1D includes inhibition of T-cell proliferation, skewing of Th cell differentiation and partial depletion of Th cells, and protection of *β* pancreatic islets.

### 3.2. Action and Mechanism of *B. pilosa* for Type 2 Diabetes

Mounting evidence from epidemiological studies proposes environmental and genetic factors as the primary causes of T2D. Both factors contribute to insulin resistance and loss of *β*-cell function, leading to impairment in insulin action, insulin production, or both. As a result, this impairment accompanies the development of hyperglycemia, a major pathological feature of T2D [26]. Hyperglycemia can cause damage to *β* cells and other peripheral tissues, named glucotoxicity. As a consequence, cardiovascular disease, nephropathy, retinal blindness, neuropathy, and peripheral gangrene develop and contribute to mortality [27]. Therefore, maintenance of glycemic homeostasis has been a golden standard for T2D therapy. Moreover, aberrant lipid metabolism in adipose and other tissues can cause lipotoxicity, which can further worsen diabetic complications. The *β* cells in the pancreas are the key players in glycemic homeostasis [28].

**Figure 5:**
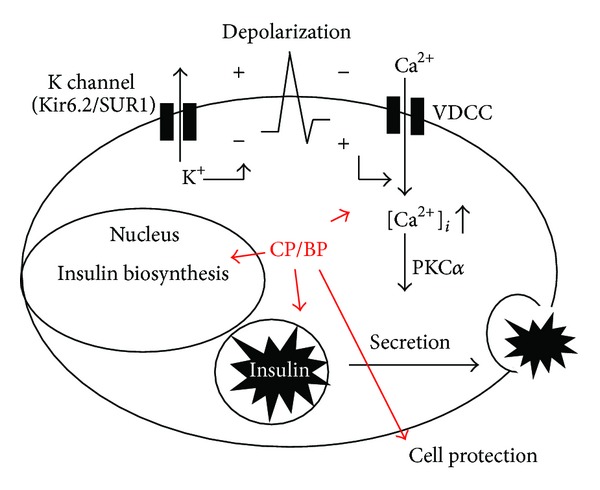
The underlying mechanism of the crude extract of *B. pilosa* (BP) and its active compound, compound **19** (CP), in T2D. BP and/or CP can treat T2D development via control of *β* cell function in db/db mice. Their anti-diabetic actions are through upregulation of insulin expression/secretion and protection of *β* cells involving secondary messengers (calcium and diacylglycerol) and their downstream PKC*α* pathway.

Plants are an extraordinary source for anti-diabetic agents. Over 1200 plant species have be claimed to treat diabetes [6, 7]. One of them, *B. pilosa*, has traditionally been used as an anti-diabetic herb in America, Africa, and Asia [7, 29, 30]. More recently, *B. pilosa* has scientifically been investigated for anti-diabetic activity. One seminal study by Ubillas et al. showed that the aqueous ethanol extract of the aerial part of* B. pilosa* at 1 g/kg BW lowered blood glucose in db/db mice, a T2D mouse model [30]. They also used a bioactivity-guided identification strategy to identify two polyynes, compounds **17** and **16**. Moreover, the mixture of the compounds (**17** : **16**) in a 2 : 3 ratio significantly reduced blood glucose level and food intake on the second day when administered at doses of 0.25 g/kg twice a day to C5BL/Ks-db/db mice. When evaluated at 0.5 g/kg, a more substantial decrease in blood glucose level as well as the stronger anorexia (food intake reduced from 5.8 g/mouse/day to 2.5 g/mouse/day) was noticed [30]. This study suggested that compounds **17** and **16** were active ingredients of *B. pilosa* for diabetes [30]. The anti-diabetic effect of both polyynes was partially caused by the hunger suppressing effect. However, the anoxic effect of the ethanol extract of *B. pilosa *was not observed in the studies described below. In another study [31], water extracts of *B. pilosa *(BPWE) were tested in diabetic db/db mice, aged 6–8 weeks, with postmeal blood glucose levels of 350 to 400 mg/dL. Like oral anti-diabetic glimepiride, which stimulates insulin release, one-single dose of BPWE reduced blood glucose levels from 374 to 144 mg/dL. The antihyperglycemic effect of BPWE was relevant to an increase in serum insulin levels, implying that BPWE drops blood glucose concentration through an upregulation of insulin production. However, BPWE showed different insulin secretion kinetics from glimepiride [31]. One drawback in current anti-diabetic agents is their decreasing efficacy over time. Therefore, the authors investigated the long-term anti-diabetic effect of BPWE in db/db mice. BPWE lowered blood glucose, boosted blood insulin, improved glucose tolerance, and reduced the percentage of glycosylated hemoglobulin (HbA1c). Both one-time and long-term experiments strongly support the superior action of BPWE on diabetes [31]. Unlike glimepiride, which failed to preserve pancreatic islets, BPWE was significantly protected against islet atrophy in mouse pancreas. The group also evaluated anti-diabetic effect of 3 *B. pilosa* varieties, *B. pilosa* var. *radiate *(BPR), *B. pilosa* var. *pilosa *(BPP), and *B. pilosa* var. *minor* (BPM) in db/db mice [15]. A single oral dose (10, 50, and 250 mg/kg BW) of BPR, BPP, or BPM water extracts decreased postprandial blood glucose levels in db/db mice for up to four hours and this reduction was dose-dependent. Of note, BPR extract resulted in a higher reduction in blood glucose levels when administered at the same dose as the other two varieties. Further, the BPR extract increased serum insulin levels in db/db mice to a greater extent than the other varieties at the same dose, 50 mg/kg. Three polyynes, compounds **16**, **17**, and **19**, were identified from the three *Bidens* strains though their varied contents. Compound **19** at 0.5 mg/kg exerted a better stimulation for insulin production in db/db mice than compounds **17** and **16**. On the contrary, 28-day treatment with the *Bidens* extracts and three polyynes were then performed using diabetic mice with postprandial glucose levels from 370 to 420 mg/dL and glimepiride was used as positive control. The applied dosages ranged from 10 mg/kg to 250 mg/kg BW. Results showed that the positive control as well as the crude extracts of the three varieties lowered the blood glucose levels in db/db mice. However, only BPR extract, containing a higher content of cytopiloyne (**19**), reduced blood glucose levels and augmented blood insulin levels more than BPP and BPM. The percentage of glycosylated hemoglobin A1c (HbA1c), a long-term indicator of blood homeostasis, was also monitored. HbA1c in the blood of 10- to 12-week-old diabetic mice was 7.9 ± 0.5%. However, treatment with BPR crude extract (50 mg/kg), glimepiride (1 mg/kg), and compound **19** (0.5 mg/kg) led to the HbA1c of 6.6 ± 0.2%, 6.1 ± 0.3%, and 6.2 ± 0.3% in the blood of age-matched mice, respectively [15]. Since cytopiloyne (**19**) was the most effective polyyne found in *B. pilosa*, against T2D, it was used for further study on anti-diabetic action and mechanism in another study [22]. The data confirmed that cytopiloyne reduced postmeal blood glucose levels, increased blood insulin, improved glucose tolerance, suppressed HbA1c level, and protected pancreatic islets in db/db mice. Nevertheless, cytopiloyne never managed to decrease blood glucose in streptozocin- (STZ-) treated mice whose *β* cells were already destroyed. In addition, cytopiloyne dose-dependently promoted insulin secretion and expression in *β* cells as well as calcium influx, diacylglycerol, and activation of protein kinase C*α*. Taken together, the mechanistic studies suggest that cytopiloyne acts to treat T2D via regulation of *β* cell functions (insulin production and *β* cell preservation) involving the calcium/diacylglycerol/PKC*α* cascade (Figure 5).

The above studies point to the conclusion that cytopiloyne (**19**) and related polyynes (compounds **16** and** 17**) are anti-diabetic agents in animal models. The data unfold a new biological action of polyynes. However, like all drugs developed for diabetes, cytopiloyne could neither prevent nor stop diabetes completely but reduced diabetic complications [22]. Intriguingly, 36 polyynes have been found in *B. pilosa* so far. It remains to be investigated whether all the polyynes present in this plant have anti-diabetic activities.

## 4. Phytochemistry

**Figure 6:**
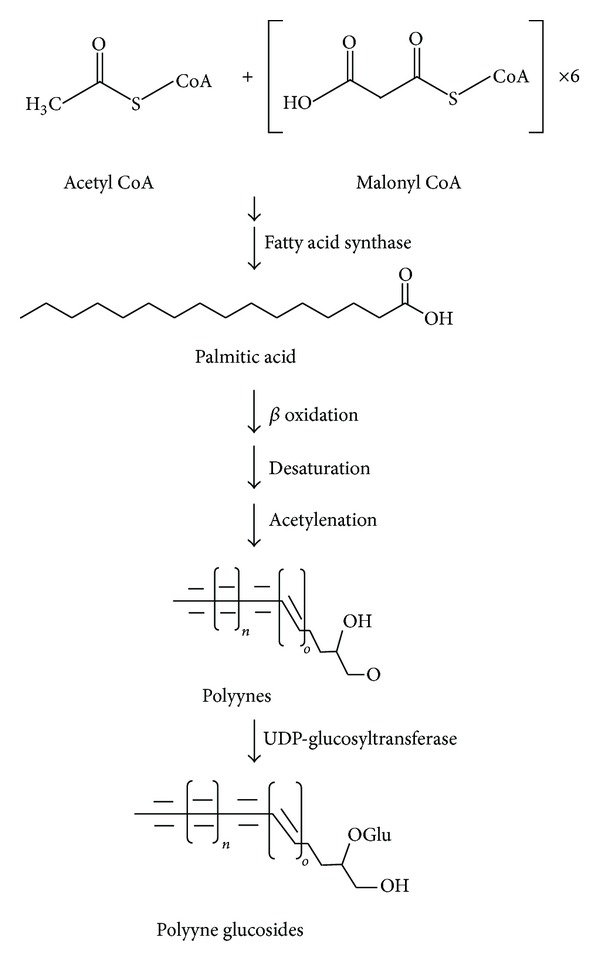
A scheme describing the biosynthesis of polyynes and its glucosides. Saturated fatty acids (e.g., palmitic acid, etc.) are synthesized from their common precursors, acetyl CoA and malonyl CoA. To generate acetylenic fatty acids (i.e., polyynes), the saturated fatty acids are proposed to undergo *β* oxidation, desaturation, and acetylenation. UDP-glucosyltransferase can attach a glucose moiety or more to polyynes to produce polyyne glucosides.

Broad application of *B. pilosa* all over the world has led to over 120 publications on its exploitation and use in medicines, foods, and drinks. *B. pilosa* is an extraordinary source of phytochemicals and 201 compounds have so far been identified from this plant, including 70 aliphatics (36 polyynes), 60 flavonoids, 25 terpenoids, 19 phenylpropanoids, 13 aromatics, 8 porphyrins, and 6 other compounds [32]. Mounting evidence suggests that phytochemical complexity of *B. pilosa* can account for its diverse bioactivities. The structures and likely bioactivities of these compounds were recently reviewed in the previous publications [5, 32], which are out of our scope. In this section, we focus on the chemical structures of 36 polyynes found in *B. pilosa* (Table 4) and their bioactivities (Table 5). We also discuss the likely biosynthesis of the polyynes in *B. pilosa*. Although their biosynthesis is not well defined, those polyynes are thought to derive from desaturation and acetylenation of fatty acids (Figure 6) in *B. pilosa* and other plants.

## 5. Toxicology 

Food and Agricultural Organization of the United Nations has reported *B. pilosa* as a staple food and promoted its cultivation in Africa since 1975. Taiwanese Department of Health also allows its use as an ingredient in food for human consumption. Despite lack of systemic toxicological study of* B. pilosa* in humans, some information about acute and/or subchronic toxicities was revealed in rodents. Frida and colleagues reported that one-single oral dose of the water extract of *B. pilosa* leaves at 10 g/kg BW showed no obvious mortality or changes in the appearance of rats [61]. The same extract at 0.8 g/kg BW/day, once a day, showed no obvious sub-chronic toxicity in rats over 28 days, as shown by survival rate, body weight, and gross examination of organs [61]. They also evaluated the acute toxicity of hydroethanol extracts of *B. pilosa* in mice [61]. Five- to six-week-old mice, weighing between 28 and 35 g, received a peritoneal injection of both extracts at the different doses. The LD_50_, the dose that causes 50% lethality, of the hydroethanol extracts in mice was 12.3 g/kg BW and 6.2 g/kg BW, respectively [61]. Ezeonwumelu et al. showed that oral delivery of the water extract of the *B. pilosa *whole plant at 1 g/kg BW/day, once a day, seems nontoxic in rats over 28 days in Wistar rats [62], which is in line with our observations in rat [5]. Overall, these data suggest that consumption of *B. pilosa *aqueous extract at up to at 1 g/kg BW/day, once a day, is highly safe in rats. A complete toxicology and drug-drug interaction of *B. pilosa* with other drugs in humans are required prior to its further medical use.

## 6. Conclusions 

*B. pilosa* is a worldwide plant and widely used as folk remedies and foods. It has long used to treat diabetes in different continents. However, a comprehensive up-to-date review of research on *B. pilosa* for diabetes has hitherto been not available. In this paper, scientific studies on the use of *B. pilosa* as an anti-diabetic remedy have been summarized and critically discussed from botanical, phytochemical, pharmacological, and toxicological aspects. Thirty-six polyynes identified from this plant were identified and three of which were showed to treat both T1D and T2D. The anti-diabetic utility of *B. pilosa* and its modes of action in relation to its known polyynes were discussed herein. Cautions should be taken in the anti-diabetic use of *B. pilosa* alone and in combination with other medicines since its overdose may cause dramatic hypoglycemia.

## Figures and Tables

**Table 1:** Taxonomy of *B*.  *pilosa* [12].

Kingdom	Plantae
Division	Magnoliophyta
Class	Magnoliopsida
Subclass	Asteridae
Order	Asterales
Family	Asteraceae
Genus	*Bidens *
Species	*Bidens pilosa* L.

**Table 2:** Percent differences between the internal transcribed spacer 1 (ITS1) regions of the DNA sequences of *B*.  *pilosa* variants (BPR, *B*.  *pilosa* var. *radiata*; BPM, *B*.  *pilosa* var. *minor*; and BPP, *B*.  *pilosa* var. *pilosa*), *B*.  *hintonii*, and *B*.  *biternata*.

ITS1	BPM	BPP	BPR	BH^a^	BB^b^
BPM	0	0.39	3.56	4.74	11.37
BPP	0.39	0	3.16	4.45	11.46
BPR	3.56	3.16	0	5.93	12.55
BH^a^	4.74	4.35	5.93	0	14.45
BB^b^	11.37	11.46	12.55	14.45	0

^a^ITS1 obtained from GeneBank Accession Number AF330101.1.

^b^ITS1 obtained from GeneBank Acession Number EU117248.1.

**Table 3:** Th1 inhibition and Th2 promotion by the extract (150 *μ*g/mL) and polyynes (10 *μ*g/mL) of *B*.  *pilosa*.

	Butanol extract	Compound **19**	Compound **17**	Compound **16**
Reduction of Th1 (%)	32%	75%	17%	9%
Increase of Th2 (%)	103%	277%	31%	6%

**Table 4:** Polyynes isolated from *B*. *pilosa* [32].

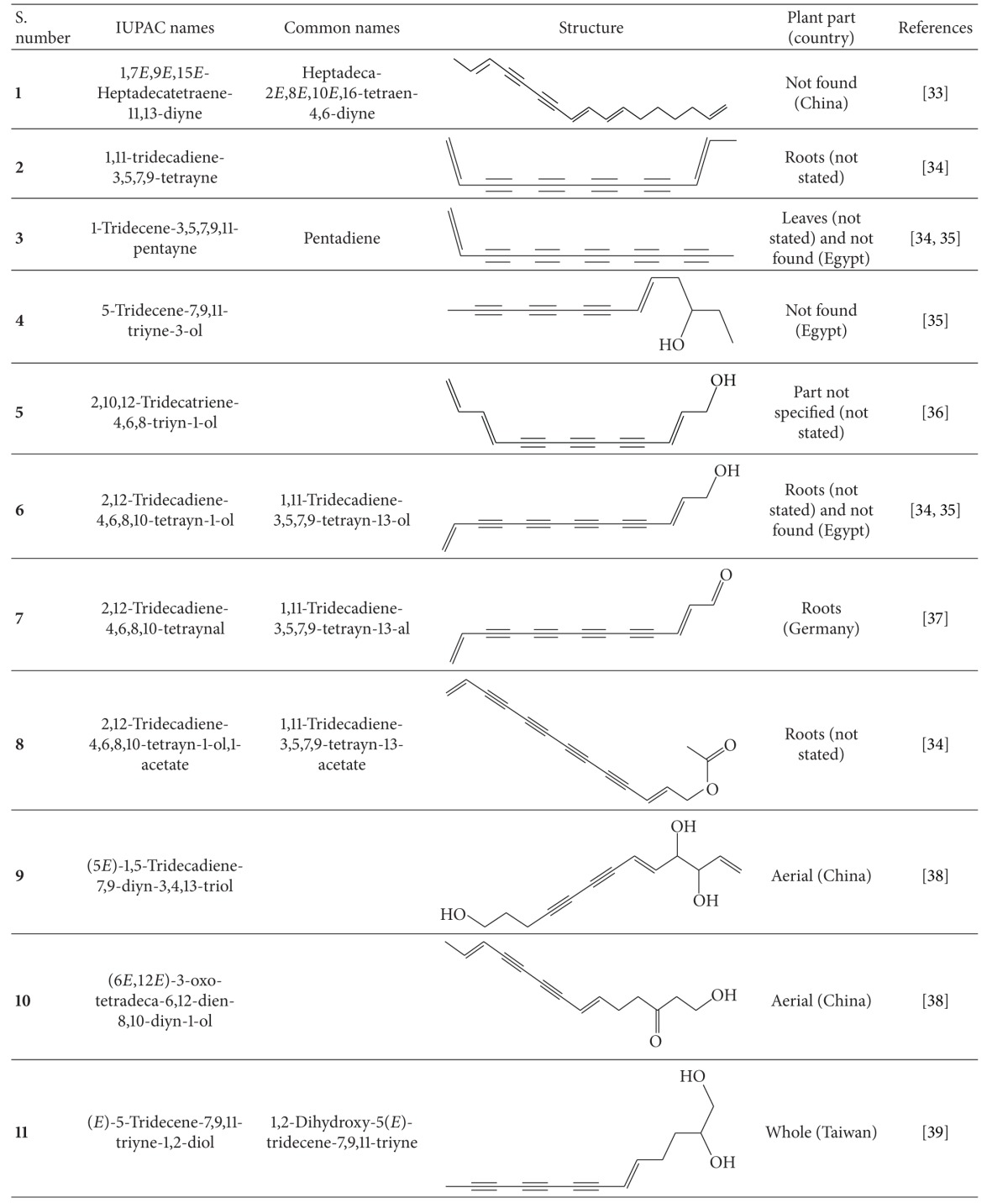 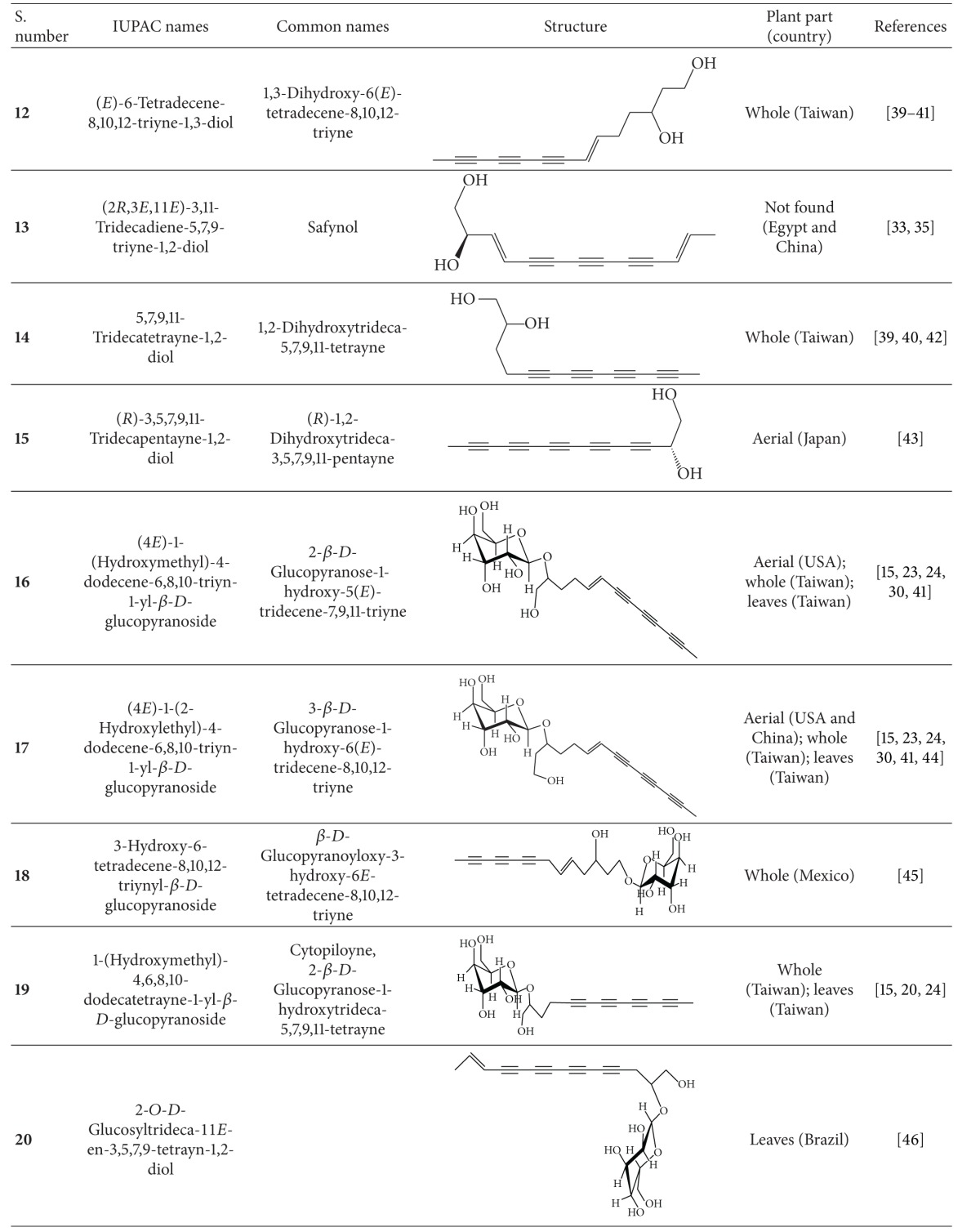 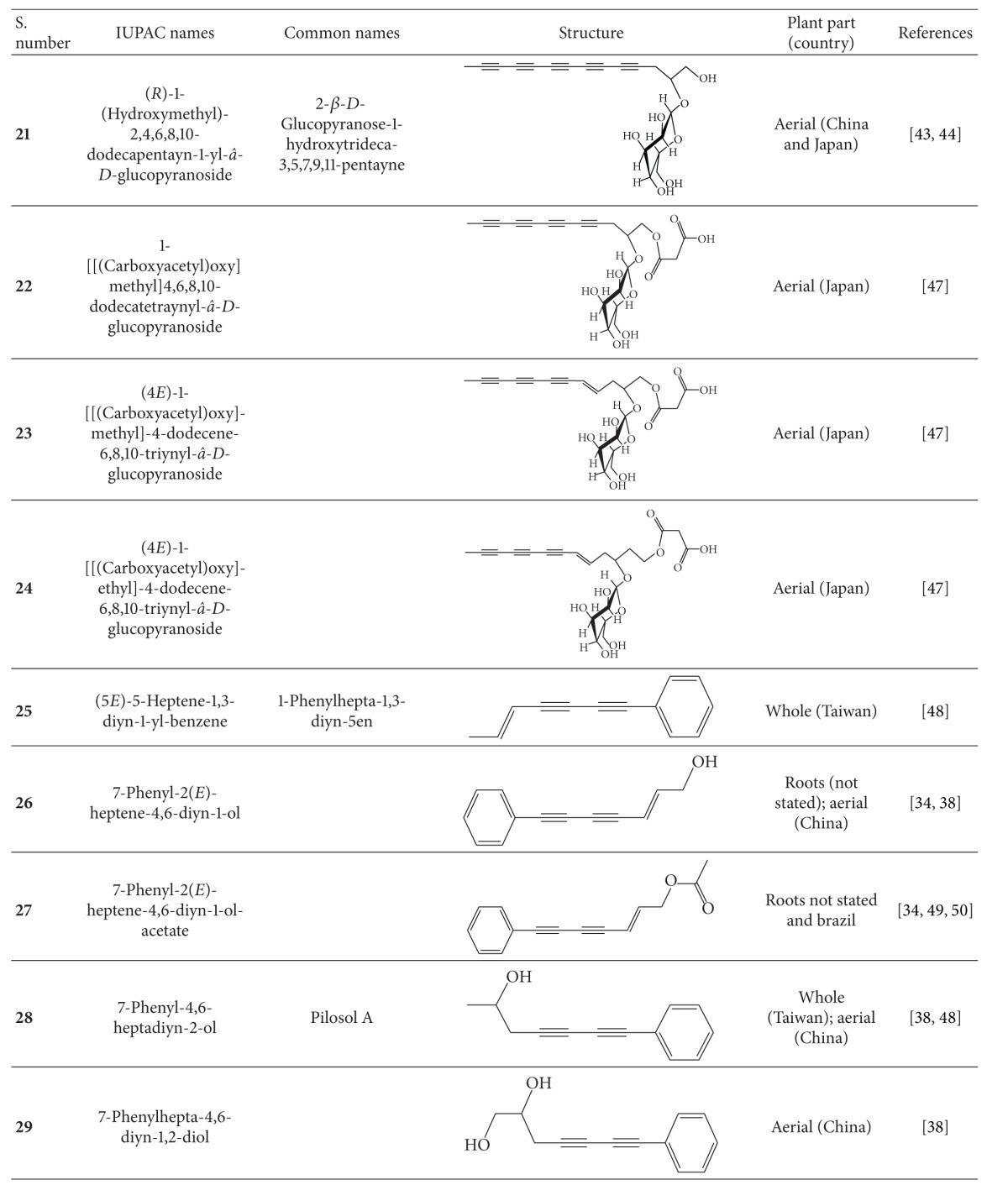 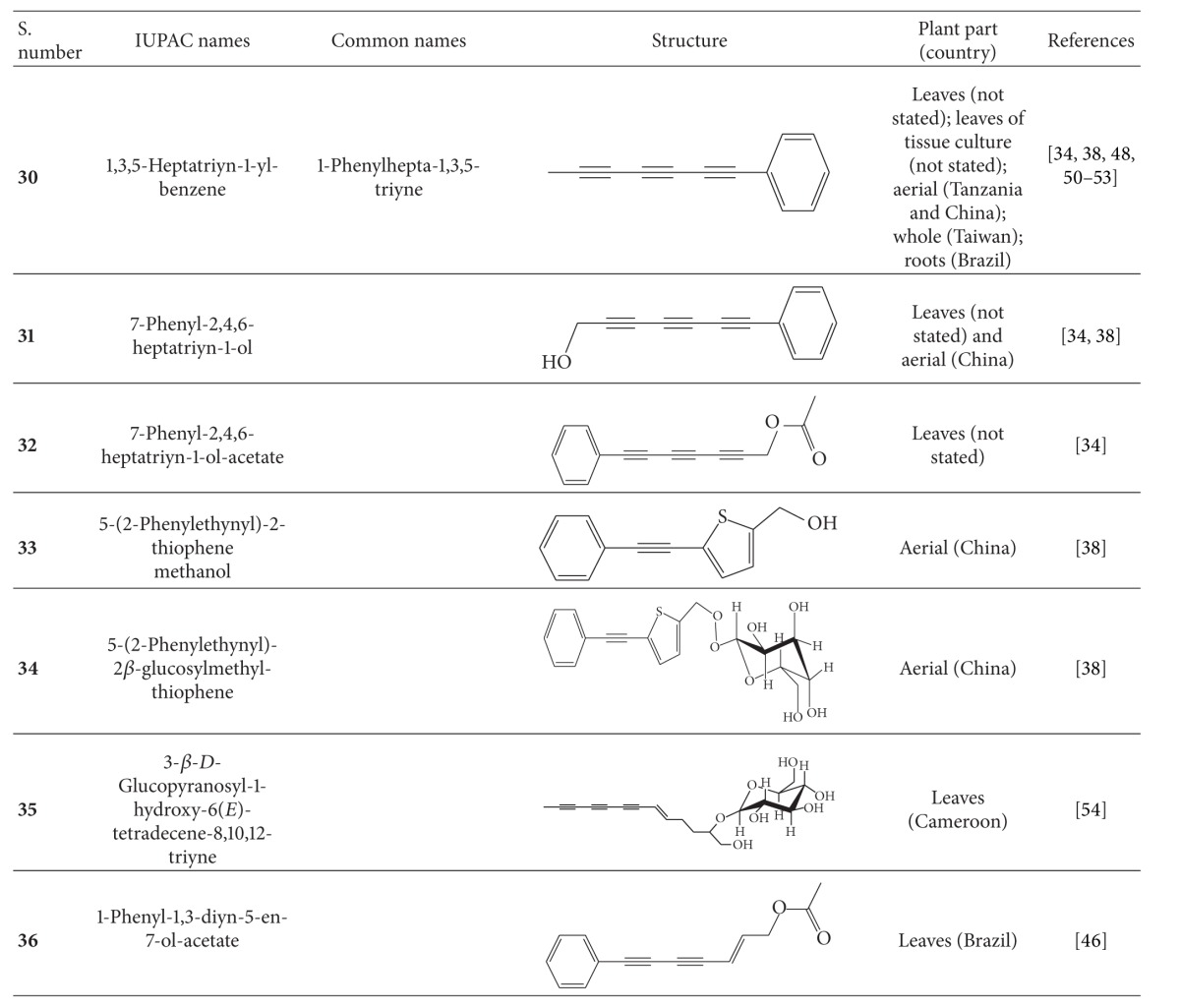

**Table 5:** Polyynes of *B*.  *pilosa* and their biological activities.

S. number	Name	Classification	Molecular formula	Biological activities
**11**	1,2-Dihydroxy-5(*E*)-tridecene-7,9,11-triyne [55]	Polyyne	C_13_H_14_O_2_	Antiangiogenetic [55]
				Antiproliferative [55]
**12**	1,3-Dihydroxy-6(*E*)-tetradecene-8,10,12-triyne [56]	Polyyne	C_14_H_16_O_2_	Antiangiogenetic [56]
**14**	1,2-Dihydroxytrideca-5,7,9,11-tetrayne [56]	Polyyne	C_13_H_12_O_2_	Antiangiogenetic [56]
**15**	(*R*)-1,2-dihydroxytrideca-3,5,7,9,11-pentayne [43]	Polyyne	C_13_H_8_O_2_	Antimalarial [43]
				Antibacterial [43]
**6**	2-*β*-*D*-Glucopyranose-1-hydroxy-5(*E*)-tridecene-7,9,11-triyne [54]	Polyyne	C_19_H_24_O_7_	Antidiabetic [54]
				Anti-inflammatory [57]
				Antimalarial [43]
				Antibacterial [43]
**30**	1-Phenylhepta-1,3,5-triyne [58]	Polyyne	C_13_H_8_	Antimicrobial [59]
				Antimalarial [9]
				Cytotoxic [9]
				Antifungal [60]
**35**	3-*β*-*D*-Glucopyranosyl-1-hydroxy-6(*E*)-tetradecene-8,10,12-triyne [54]	Polyyne	C_20_H_26_O_7_	Antidiabetic [54]
				Anti-inflammatory [57]
